# Different impacts of *TP53* mutations on cell cycle-related gene expression among cancer types

**DOI:** 10.1038/s41598-023-32092-8

**Published:** 2023-03-24

**Authors:** Keiju Sasaki, Shin Takahashi, Kota Ouchi, Yasufumi Otsuki, Shonosuke Wakayama, Chikashi Ishioka

**Affiliations:** 1 Department of Clinical Oncology, Graduate School of Medicine, Tohoku University, Sendai, Miyagi Japan; 2 Department of Medical Oncology, Tohoku University Hospital, Sendai, Miyagi Japan; 3 Department of Clinical Oncology, Institute of Development, Aging and Cancer, Tohoku University, Sendai, Miyagi Japan

**Keywords:** Gene expression, Gene regulation, Cancer genomics

## Abstract

Functional properties caused by *TP53* mutations are involved in cancer development and progression. Although most of the mutations lose normal p53 functions, some of them, gain-of-function (GOF) mutations, exhibiting novel oncogenic functions. No reports have analyzed the impact of *TP53* mutations on the gene expression profile of the p53 signaling pathway across cancer types. This study is a cross-cancer type analysis of the effects of *TP53* mutations on gene expression. A hierarchical cluster analysis of the expression profile of the p53 signaling pathway classified 21 cancer types into two clusters (A1 and A2). Changes in the expression of cell cycle-related genes and *MKI67* by *TP53* mutations were greater in cluster A1 than in cluster A2. There was no distinct difference in the effects between GOF and non-GOF mutations on the gene expression profile of the p53 signaling pathway.

## Introduction

*TP53*, encoding p53, is the most frequently mutated gene in human tumors^1–3^, and it is estimated that > 50% of all human cancers show mutations^2,4^. p53 regulates the expression of downstream genes as a transcriptional activating factor through specific DNA binding sites^5^. Its most well-known functions include anti-proliferative effects, such as cell cycle arrest^6–8^, DNA repair^9,10^, apoptosis^11^, and cellular senescence^1–3^. Additionally, p53 induces cell death by apoptosis for irreparable DNA damage^11^. During DNA damage, the activated p53 promotes DNA repair by inhibiting cell proliferation and arresting the cell cycle. It also prevents the accumulation of genetic mutations by eliminating unrepairable cells. Therefore, p53 is called “the guardian of the genome”^11^. p53 reportedly suppresses the IGF-1/mTOR pathway^12^ and enhances antitumor effects through exosome secretion^13^.

The category classifications and gene lists based on each function of p53 can be reviewed in the “Kyoto Encyclopedia of Genes and Genomes” (KEGG, http://www.genome.jp/kegg/). KEGG is an integrated bioinformatics database that links genomes with various functional information including pathways (interaction networks between molecules in metabolic and signaling pathways), and continues to be manually developed and updated based on literature information^14–16^. Information on various KEGG pathways, including the p53 signaling pathway, may be used for pathway analyses and enrichment analyses. In fact, as of February 1, 2023, a PubMed search for “kegg” and “pathway analysis” yielded 3,561 reports from 2005 to 2023 (2,007 reports from 2020 to 2023), and the search for “kegg” and “enrichment analysis” yielded 5,725 reports from 2006 to 2023 (4,370 reports from 2020 to 2023). This indicates that KEGG pathway database is recognized as an important database even after 2020. The p53 signaling pathway in the KEGG pathway database (https://www.genome.jp/kegg/pathway.html) lists 67 genes as genes on the p53 signaling pathway in September 2021. In the category classification, the genes are first classified into categories of genes upstream and downstream of the p53 signaling pathway. The downstream gene categories are further classified by their functions into apoptosis-, cell cycle-, DNA repair and damage prevention-, exosome secretion-, angiogenesis and metastasis formation suppression-, IGF-1/mTOR pathway suppression-, and p53 negative feedback-related categories.p21 is required for examining the functional activity of p53. p21 is a protein downstream of the p53 signaling pathway and is involved in cell cycle inhibition and cellular senescence through p53-dependent and -independent pathways^17,18^. Since p21 activity is greatly affected by the regulation of its expression by p53^7^, it is considered a useful surrogate marker when examining the functional activity of p53. The International Agency for Research on Cancer (IARC, https://www.iarc.who.int/) database has published sequence-specific transcriptional activities of mutant p53 of all 2341 variants of mutant p53 with one amino acid substitution for p21, MDM2, BAX, 14-3-3σ, AIP1, GADD45, NOXA and p53R2^19^. The *TP53* database was transferred from IARC to US National Cancer Institute on October 25, 2021. They are now available on https://tp53.isb-cgc.org/.

The mutations in the *TP53* gene result in loss of normal p53 functions such as senescence and apoptosis, and affect the carcinogenic process^20^. *TP53* mutations have been reported as a poor prognostic factor in breast cancer^21^, head and neck cancer^22^, and hematologic malignancies^23^. Contrarily, there are many reports showing no association between *TP53* mutations and prognosis in cancers, such as colon^24^, lung^25^, and bladder cancers^26^. Several reports suggest that *TP53* mutations are a favorable prognostic factor in brain tumors^27^ and ovarian cancer^28^; thus, the prognostic impact of *TP53* mutations may differ by cancer type^29^. Additionally, *TP53* mutations have been reported to be useful as a marker of response to chemotherapy in lung^30^ and breast cancers^31^. Contrarily, they have also been found to be a marker of refractory to chemotherapy in head and neck cancers^32^, suggesting that the effects of *TP53* mutations on drug sensitivity may also differ by cancer type^29^. However, no report to date has analyzed these points across different cancer types.

The majority of *TP53* mutations are missense mutations. Some studies have shown that some missense mutations in p53 not only deactivate the tumor suppressor function of p53, but also acquire novel oncogenic functions such as tumor cell proliferation, antiapoptotic effects, and promotion of angiogenesis and metastasis formation. These are termed gain-of-function (GOF) mutations, a subtype of *TP53* mutations^4,33–36^. The concept of GOF mutations is well established, but no clear definition has been established so far^37^. Since GOF is a mutation that gains a new function as described above in addition to the loss of p53 function, it is not a simple counterpart of a loss-of-function mutation^37^. GOF mutations are more likely to accumulate p53 in the nucleus than non-gain-of-function (non-GOF) mutations, and they induce extensive loss of transcriptional function. Contrarily, many non-GOF mutations have been reported to preserve a certain level of transcriptional function and to maintain normal activation of p53-regulated genes to some degrees^37,38^. Furthermore, GOF mutations have been reported to be a possible poor prognostic factor in glioblastomas^39^ and T-cell lymphomas^40^. The differences in *TP53* mutation subtypes (e.g., GOF vs. non-GOF mutations) may have different effects on other gene expressions in different cancer types^41,42^. However, there have been no reports comparing these differences across cancer types.

Based on this background, we hypothesized that the changes in the gene expression profile of the p53 signaling pathway caused by *TP53* mutations differ among cancer types. We also hypothesized that the effects of different *TP53* mutation subtypes (GOF vs. non-GOF mutations) on the gene expression profile of the p53 signaling pathway would differ by cancer type. Then, we verified these hypotheses by comparing the changes in the gene expression profile of the p53 signaling pathway caused by *TP53* mutations across cancer types.

## Results

**Figure 1:**
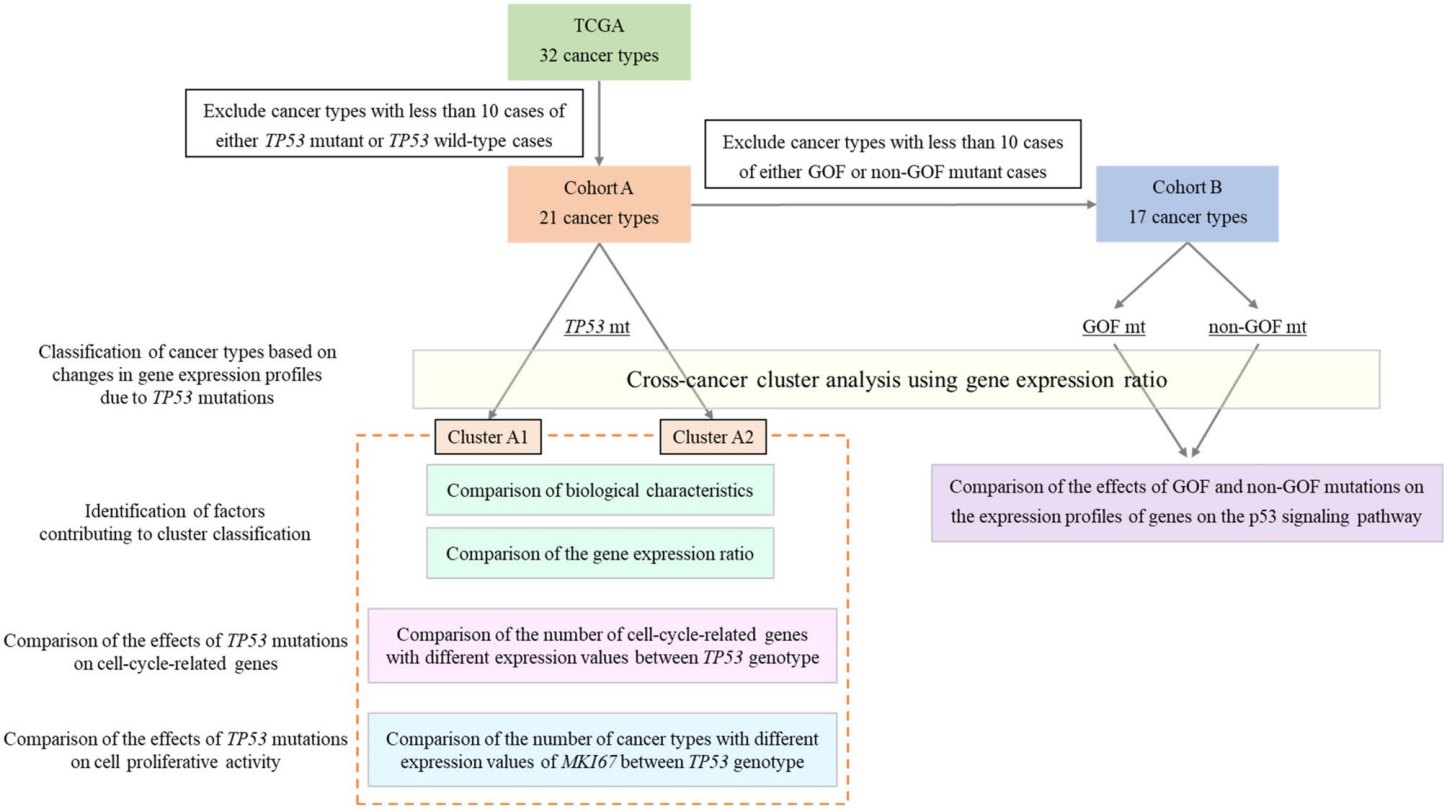
The workflow of analyses. TCGA, The Cancer Genome Atlas; *TP53* mt, *TP53* mutation; *TP53* wt, *TP53* wild-type; GOF mt, *TP53* gain-of-function mutation; non-GOF mt, *TP53* non-gain-of-function mutation.

The study workflow is shown in Fig. 1.

### Comparison of p53 transcriptional activity

The transcriptional activity of mutant p53 on p21 was significantly correlated with that of mutant p53 on all other target genes (all *P* < 0.0001, r = 0.70–0.83, Supplementary Tables S1 and S2).

### Cancer types for analyses

The cases of all 32 cancer types listed in The Cancer Genome Atlas (TCGA, https://portal.gdc.cancer.gov/) database were classified into two groups according to the presence or absence of *TP53* mutations (Table 1). As a result, 21 cancer types (adrenocortical carcinoma, bladder urothelial carcinoma, breast invasive carcinoma, cervical squamous cell carcinoma, colon adenocarcinoma, esophageal carcinoma, glioblastoma multiforme, head and neck squamous cell carcinoma, kidney chromophobe, brain lower grade glioma, liver hepatocellular carcinoma, lung adenocarcinoma, lung squamous cell carcinoma, mesothelioma, ovarian serous cystadenocarcinoma, pancreatic adenocarcinoma, prostate adenocarcinoma, rectum adenocarcinoma, sarcoma, stomach adenocarcinoma, and uterine corpus endometrial carcinoma) contained 10 or more cases each of *TP53* mutant and wild-type cases. This cohort of cancer types was defined as “Cohort A.” In cohort A, the number of *TP53* mutant cases was 2,735, whereas the number of *TP53* wild-type cases was 3,618. The *TP53* mutations identified in this study consisted of 2257 (71.8%) missense, 452 (14.4%) nonsense, and 433 (13.8%) frameshift mutations. For each cancer type in cohort A, *TP53* mutant cases were further classified into GOF and non-GOF mutant cases (Supplementary Table S3). There were 1,169 (37.2%) GOF and 1973 (62.8%) non-GOF mutations (Supplementary Table S4). As a result, 17 cancer types (bladder urothelial carcinoma, breast invasive carcinoma, cervical squamous cell carcinoma, colon adenocarcinoma, esophageal carcinoma, glioblastoma multiforme, head and neck squamous cell carcinoma, brain lower grade glioma, liver hepatocellular carcinoma, lung adenocarcinoma, lung squamous cell carcinoma, ovarian serous cystadenocarcinoma, pancreatic adenocarcinoma, prostate adenocarcinoma, rectum adenocarcinoma, sarcoma, and stomach adenocarcinoma) contained 10 or more GOF mutant and non-GOF mutant cases each, and we defined this cohort of cancer types as “Cohort B.”

**Table 1:** Histological type, developmental origin, *TP53* mutation rate, GOF mutation rate, and cluster classification for each cancer type in cohort A.

Cancer type	Histlogical type	Developmental origin	Ratio of *TP53* mt (%)	Ratio of GOF (%)	Cluster classification
ACC	Non-adenocarcinoma	Mesoderm	15.9	9.1	A1
BLCA	Non-adenocarcinoma	Endoderm	47.0	41.3	A1
BRCA	Adenocarcinoma	Ectoderm	31.0	34.2	A1
ESCA	Adenocarcinoma	Endoderm	86.1	46.6	A1
GBM	Non-adenocarcinoma	Ectoderm	31.4	53.5	A1
KICH	Non-adenocarcinoma	Mesoderm	24.6	21.4	A1
LGG	Non-adenocarcinoma	Ectoderm	46.4	61.1	A1
LIHC	Non-adenocarcinoma	Endoderm	27.5	42.2	A1
LUAD	Adenocarcinoma	Endoderm	44.7	23.2	A1
LUSC	Non-adenocarcinoma	Endoderm	83.0	32.6	A1
MESO	Non-adenocarcinoma	Mesoderm	15.1	23.1	A1
OV	Adenocarcinoma	Mesoderm	94.1	35.9	A1
PAAD	Non-adenocarcinoma	Endoderm	49.6	42.9	A1
PRAD	Adenocarcinoma	Mesoderm	9.5	32.6	A1
SARC	Non-adenocarcinoma	Mesoderm	32.5	32.8	A1
STAD	Adenocarcinoma	Endoderm	43.7	38.3	A1
UCEC	Adenocarcinoma	Mesoderm	35.1	49.7	A1
CESC	Non-adenocarcinoma	Mesoderm	7.7	19.0	A2
COAD	Adenocarcinoma	Endoderm	52.0	48.5	A2
HNSC	Non-adenocarcinoma	Ectoderm	66.3	11.2	A2
READ	Adenocarcinoma	Endoderm	71.4	54.4	A2

### Genes on the p53 signaling pathway for analyses

The 67 genes registered as genes on the p53 signaling pathway and category classification defined by KEGG for each gene are listed in Supplementary Table S5. For these genes, the median expression of each gene was calculated for each cancer type. The median expression of each gene and the number of cancer types for which the median expression was less than the expression threshold (Transcripts Per Million (TPM) value 1.0^43^) are shown in Supplementary Table S6. Genes whose median expression was below the threshold in more than half of the cancer types were common in cohorts A and B (*ADGRB1*, *IGF1*, *RPRM*, *TP53AIP1*). Therefore, 63 genes were selected for analyses in this study.

### A cross-cancer unsupervised hierarchical cluster analysis based on changes in the gene expression profile of the p53 signaling pathway

**Figure 2:**
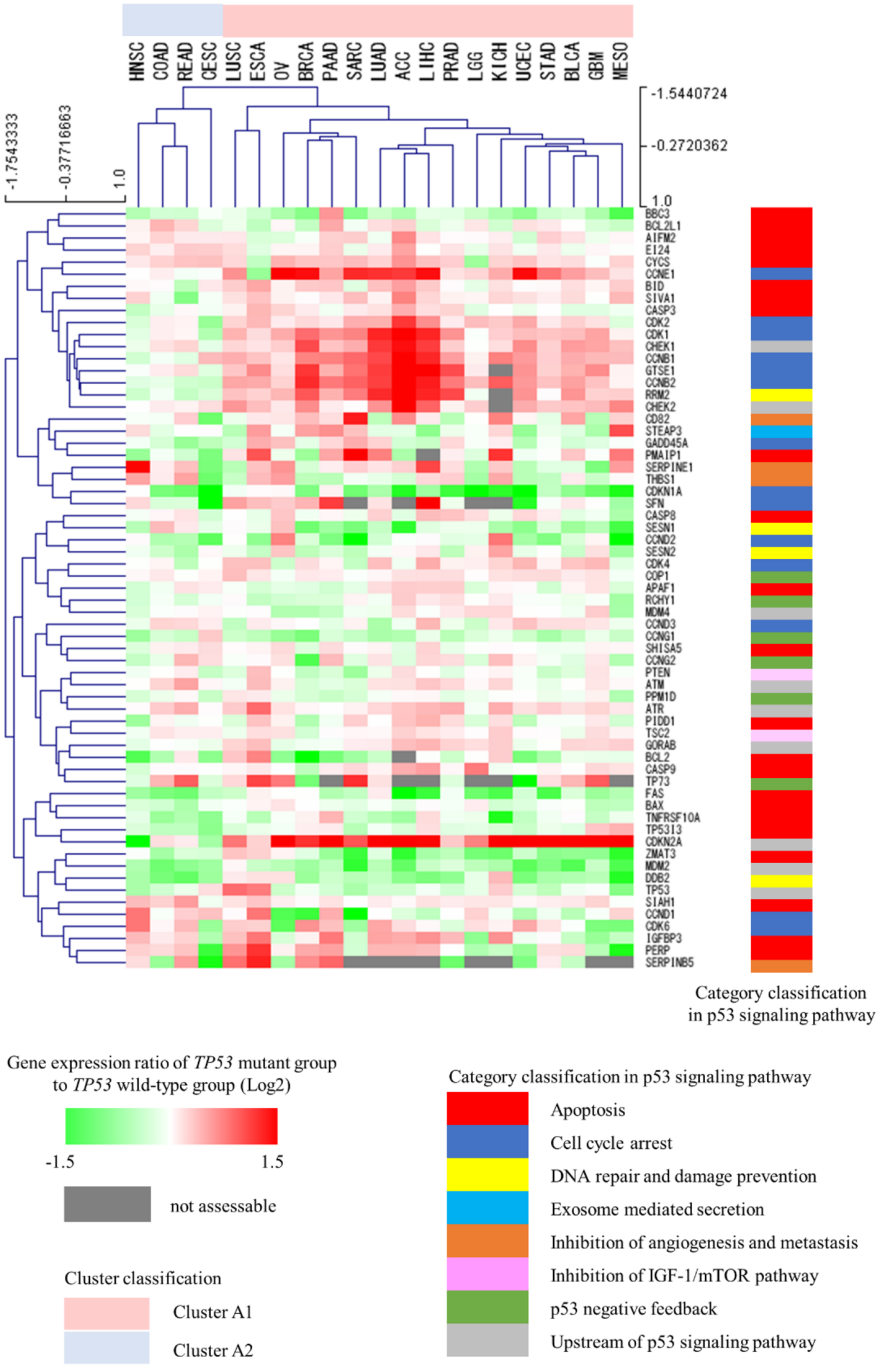
Unsupervised cross-cancer hierarchical clustering. This cluster analysis was performed using the complete linkage method with Pearson correlation distance. *ACC* Adrenocortical carcinoma, *BLCA* Bladder urothelial carcinoma, *BRCA* Breast invasive carcinoma, *CESC* Cervical squamous cell carcinoma, *COAD* Colon adenocarcinoma, *ESCA* Esophageal carcinoma, *GBM* Glioblastoma multiforme, *HNSC* Head and neck squamous cell carcinoma, *KICH* Kidney chromophobe, *LGG* Brain lower grade glioma, *LIHC* Liver hepatocellular carcinoma, *LUAD* Lung adenocarcinoma, *LUSC* Lung squamous cell carcinoma, *MESO* Mesothelioma, *OV* Ovarian serous cystadenocarcinoma, *PAAD* Pancreatic adenocarcinoma, *PRAD* Prostate adenocarcinoma, *READ* Rectum adenocarcinoma, *SARC* Sarcoma, *STAD* Stomach adenocarcinoma, *UCEC* Uterine corpus endometrial carcinoma.

To examine whether the changes in the gene expression profile of the p53 signaling pathway caused by *TP53* mutations differ among cancer types, we performed a cross-cancer unsupervised hierarchical cluster analysis using the gene expression ratios of the p53 signaling pathway in cohort A. As a result, the 21 cancer types in cohort A were classified into clusters comprising 17 (cluster A1) and 4 (cluster A2) cancer types. Cluster A1 included adrenocortical carcinoma, bladder urothelial carcinoma, breast invasive carcinoma, esophageal carcinoma, glioblastoma multiforme, kidney chromophobe, brain lower grade glioma, liver hepatocellular carcinoma, lung adenocarcinoma, lung squamous cell carcinoma, mesothelioma, ovarian serous cystadenocarcinoma, pancreatic adenocarcinoma, prostate adenocarcinoma, sarcoma, stomach adenocarcinoma, and uterine corpus endometrial carcinoma, whereas cluster A2 included cervical squamous cell carcinoma, colon adenocarcinoma, head and neck squamous cell carcinoma, and rectum adenocarcinoma (Fig. 2).

### Identification of factors contributing to cluster A1 and A2 classifications

We explored the factors contributing to the classification of clusters A1 and A2. The histological type, developmental origin, *TP53* mutation rate, GOF mutation rate, and cluster classification for each cancer type are shown in Table 1. An examination of the histological bias between the two clusters revealed no statistically significant difference in the proportion of adenocarcinoma and non-adenocarcinoma (*P* = 1.0000, Supplementary Table S7). The same study of developmental origin bias found no significant difference between the two clusters (*P* = 1.0000, Supplementary Table S8). We also examined whether there was any bias in the proportion of *TP53* genotype between the two clusters and found no statistically significant differences in either the proportion of *TP53* (*P* = 0.4737) or GOF (*P* = 0.7055) mutant cases. We additionally examined whether the proportion of hotspot mutation differed between clusters A1 and A2, but there were no significant differences (*P* = 0.6945, Supplementary Table S9).

The expression ratios of each gene on the p53 signaling pathway were compared between clusters A1 and A2. Fifteen genes had significantly different gene expression ratios between the two clusters (Supplementary Table S10). These extracted genes were classified by using the KEGG category: six genes were constitutive for the cell cycle-related category, and four genes each were constitutive for the category upstream of the p53 signaling pathway and for apoptosis-related category. In each of these categories, the extracted genes accounted for 42.9% (6/14) of the genes related to the cell cycle, 44.4% (4/9) of the genes upstream of the p53 signaling pathway, and 17.3% (4/23) of the genes related to apoptosis (Table 2).

**Table 2:** Results of comparison of gene expression between cluster A1 and A2 (counted for each category classification).

Category classification in the KEGG p53 pathway	Number of component genes	Number of genes with significant differences between cluster A1 and A2 (%)
Cell cycle arrest	14	6 (42.9)
Upstream of p53 signaling pathway	9	4 (44.4)
Apoptosis	23	4 (17.3)
DNA repair and damage prevention	4	1 (25.0)
Inhibition of angiogenesis and metastasis	4	0 (0)
Inhibition of IGF-1/mTOR pathway	2	0 (0)
p53 negative feedback	6	0 (0)
Exosome mediated secretion	1	0 (0)
Total	63	15

### Comparison of expression values of cell cycle-related genes in each cancer type in cohort A

Based on the results obtained from the analyses described so far, it was suggested that cell proliferative activity might be important as a phenotypic difference between clusters A1 and A2 caused by *TP53* mutations. We, therefore, compared the expression values of 14 cell cycle-related genes on the p53 signaling pathway between the *TP53* mutant and *TP53* wild-type groups for each cancer type. The number of genes with significantly different expression values between the two groups was significantly higher in the 17 cancer types in cluster A1 than in the four cancer types in cluster A2 (*P* = 0.0429, Supplementary Table S11).

### Comparison of *MKI67* expression values in each cancer type in cohort A

**Figure 3:**
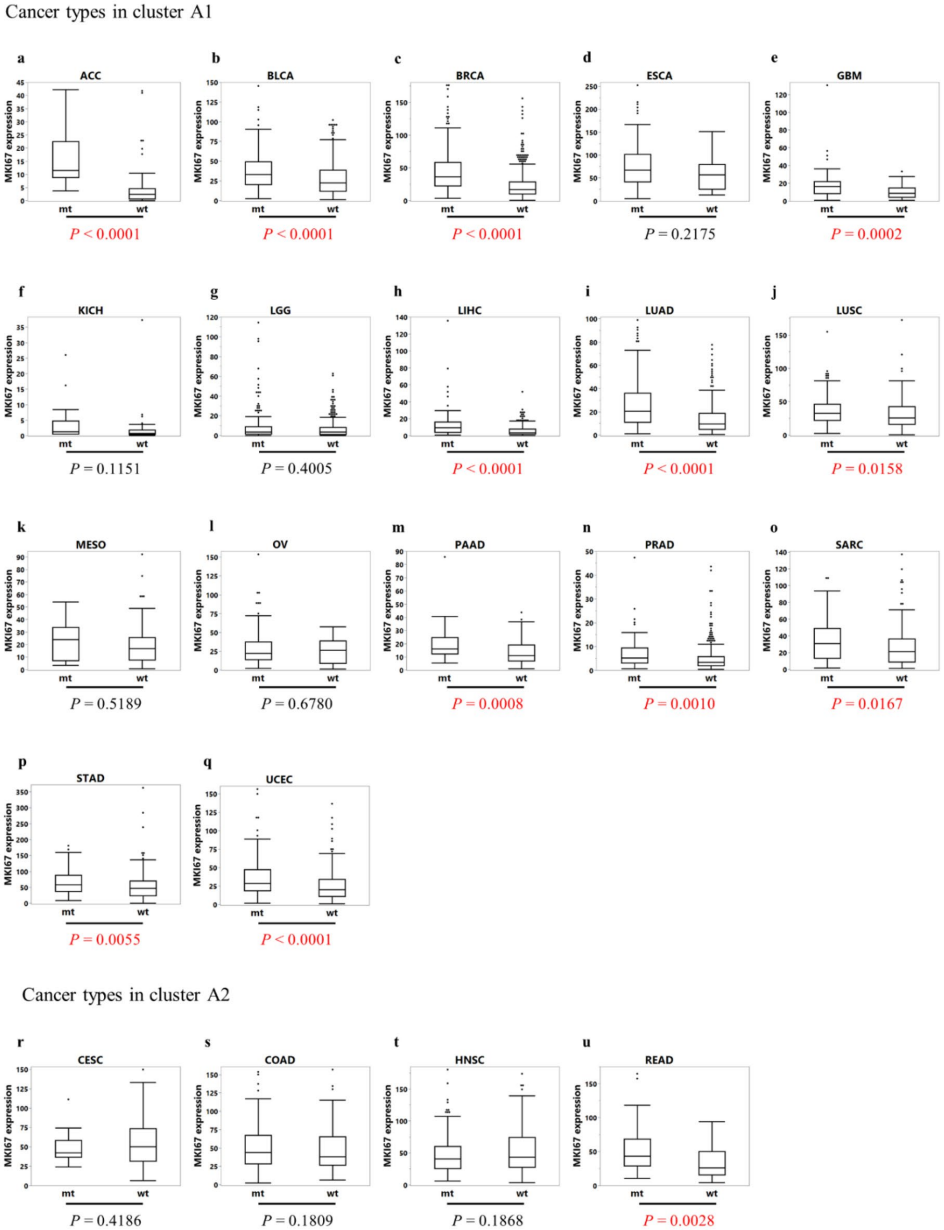
Box plot showing a comparison of the *MKI67* expression values between the *TP53* mutant and *TP53* wild-type groups for each cancer type in cohort A. Each *P*-value was calculated using the Wilcoxon rank sum test. (**a**) *ACC* Adrenocortical carcinoma, (**b**) *BLCA* Bladder urothelial carcinoma, (**c**) *BRCA* Breast invasive carcinoma; (**d**) *ESCA* Esophageal carcinoma, (**e**) *GBM* Glioblastoma multiforme, (**f**) *KICH* Kidney chromophobe, (**g**) *LGG* Brain lower grade glioma, (**h**) *LIHC* Liver hepatocellular carcinoma, (**i**) *LUAD* Lung adenocarcinoma, (**j**) *LUSC* Lung squamous cell carcinoma, (**k**) *MESO* Mesothelioma, (**l**) *OV* Ovarian serous cystadenocarcinoma, (**m**) *PAAD* Pancreatic adenocarcinoma, (**n**) *PRAD* Prostate adenocarcinoma, (**o**) *SARC* Sarcoma, (**p**) *STAD* Stomach adenocarcinoma, (**q**) *UCEC* Uterine corpus endometrial carcinoma, (**r**) *CESC* Cervical squamous cell carcinoma, (**s**) *COAD* Colon adenocarcinoma, (**t**) *HNSC* Head and neck squamous cell carcinoma, (**u**) *READ* Rectum adenocarcinoma, *mt TP53* mutant group, *wt TP53* wild-type group.

The gene expression value of *MKI67*, a marker reflecting cell proliferative activity^44,45^, was compared between the *TP53* mutant and *TP53* wild-type groups in each cancer type (Fig. 3a–u). The number of cancer types with significantly upregulated *MKI67* expression levels in the *TP53* mutant group in comparison to the *TP53* wild-type group was 12/17 (70.6%) in cluster A1 and 1/4 (25.0%) in cluster A2 (*P* = 0.2528, Table 3).

**Table 3:** Comparison of the number of cancer types with a significant difference in *MKI67* expression by *TP53* genotype between cluster A1 and A2.

Is there a significant difference in *MKI67* expression between *TP53* mt and wt?				
	Number of applicable cancer types (%)		Total	*P* value
	Yes	No		
Cluster A1	12 (70.6)	5 (29.4)	17	0.2528
Cluster A2	1 (25.0)	3 (75.0)	4	
Total	13 (61.9)	2 (38.1)	21	

### A cross-cancer unsupervised hierarchical cluster analysis based on the changes in the gene expression profile of the p53 signaling pathway caused by *TP53* mutation subtypes

**Figure 4:**
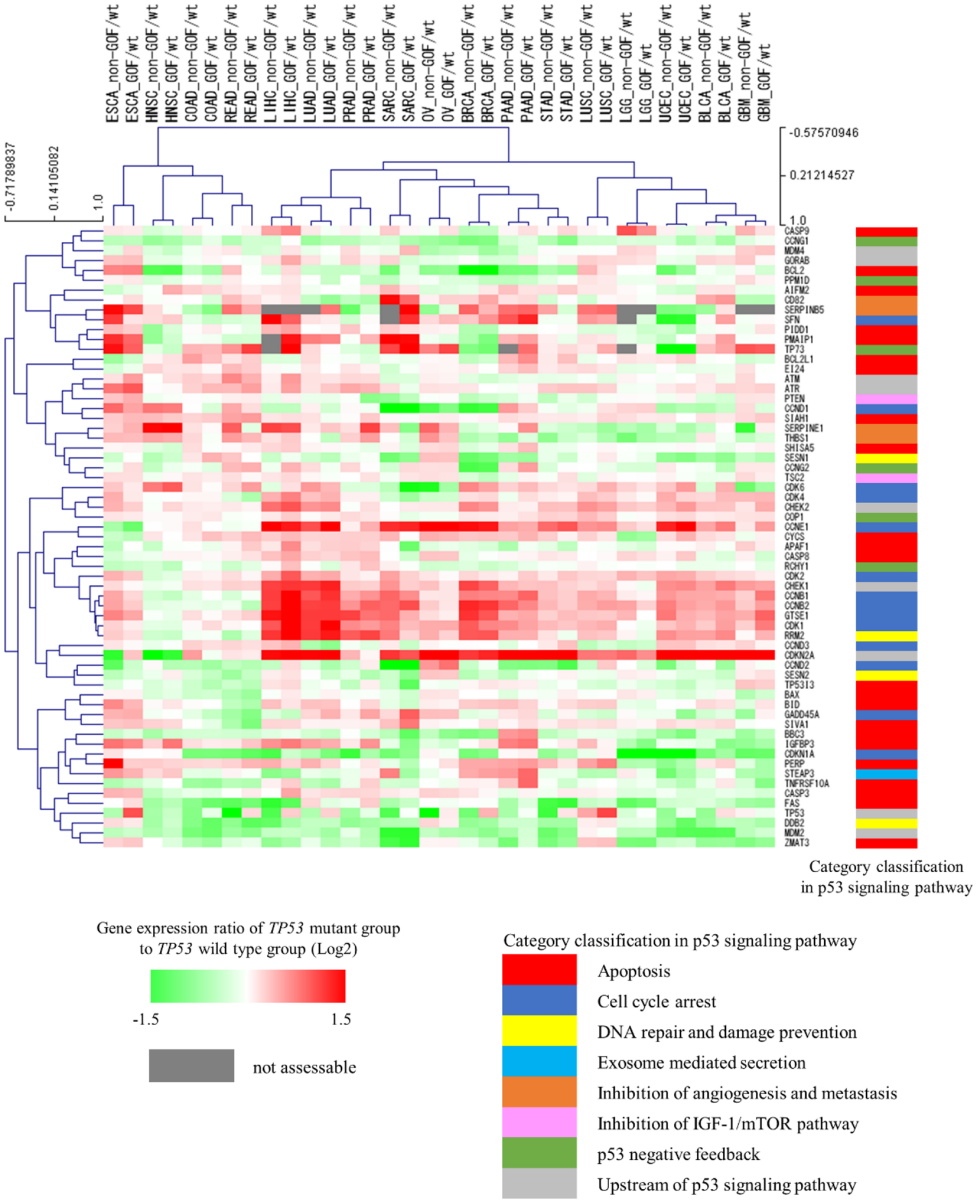
Unsupervised cross-cancer hierarchical clustering. This cluster analysis was performed using the complete linkage method with Pearson correlation distance. *BLCA* Bladder urothelial carcinoma, *BRCA* Breast invasive carcinoma, *COAD* Colon adenocarcinoma, *ESCA* Esophageal carcinoma, *GBM* Glioblastoma multiforme, *HNSC* Head and neck squamous cell carcinoma, *LGG* Brain lower grade glioma, *LIHC* Liver hepatocellular carcinoma, *LUAD* Lung adenocarcinoma, *LUSC* Lung squamous cell carcinoma, *OV* Ovarian serous cystadenocarcinoma, *PAAD* Pancreatic adenocarcinoma, *PRAD* Prostate adenocarcinoma, *READ* Rectum adenocarcinoma, *SARC* Sarcoma, *STAD* Stomach adenocarcinoma, *UCEC* Uterine corpus endometrial carcinoma, *GOF/wt* Gene expression ratio (Log2) of *TP53* gain-of-function mutant group to *TP53* wild-type group, *non-GOF/wt* Gene expression ratio (Log2) of *TP53* non-gain-of-function mutant group to *TP53* wild-type group.

To examine whether GOF and non-GOF mutations have different effects on the gene expression profile of the p53 signaling pathway, the gene expression ratios of the GOF mutant group to the *TP53* wild-type group and the non-GOF mutant group to the *TP53* wild-type group were calculated for each gene by cancer types in cohort B. Then, we performed a cross-cancer unsupervised hierarchical cluster analysis using the calculated gene expression ratios. The results showed that GOF and non-GOF mutations of the same cancer type were placed nearest to each other in all 17 cancer types (Fig. 4).

## Discussion

In this study, we examined the effects of *TP53* mutations on the gene expression profile of the p53 signaling pathway and cell proliferative activity across cancer types. The results indicated that the cancer types can be classified into two major groups based on the magnitude of gene expression changes related to the cell cycle and cell proliferative activity caused by *TP53* mutations. Furthermore, there was no distinct difference in the effects of GOF and non-GOF mutations on the gene expression profile of the p53 signaling pathway.

Because mutant p53 transcriptional activity on p21 correlates very highly with the transcriptional activity of mutant p53 on each of the other genes, the transcriptional activity on p21 was used to define the *TP53* genotype. Genes in the p53 signaling pathway listed in KEGG do not include all genes associated with p53; however, because this gene set contains the primary functions of p53 and the major genes involved in this pathway, we believe that it is reasonable to use them for assessing gene expression changes caused by *TP53* gene mutations. In addition, the use of an existing gene set eliminates selection bias during the selection of genes for analysis. Furthermore, the phenotypic changes caused by *TP53* mutations are not solely defined by the expression of genes directly downstream of *TP53* gene, but are also likely to be affected by the expression levels of genes further downstream. This is also applicable to upstream genes that receive negative feedback from p53 and their related genes. Moreover, changes in the expression of the *TP53* gene itself caused by mutations may differ among the various cancer types. For these reasons, we considered it necessary to include not only the genes directly regulated by p53 but also downstream and related genes in our analysis, which resulted in the use of the entire KEGG gene set for the p53 signaling pathway. A cross-cancer unsupervised hierarchical cluster analysis was performed to determine whether the changes in the gene expression profile of the p53 signaling pathway due to *TP53* mutations differ among cancer types. We found that the 21 cancer types in cohort A could be classified into two major clusters (A1 and A2) based on the differential effects of *TP53* mutations on the gene expression profile of the p53 signaling pathway. These two clusters were not characterized by the differences in histological type, developmental origin, *TP53* genotype proportion, the proportion of hotspot mutations. There have been several reports on the relationship between the type of GOF and the tumor spectrum in which they occur^46,47^. The results of the present study were consistent with previous reports, with a higher percentage of GOF with hotspot mutations, R249 in LIHC^48,49^ and R273 in LGG^50,51^. As genes for which gene expression changes due to *TP53* mutations contribute to cluster A1 and A2 classifications, 15 genes were extracted, consisting mainly of cell cycle-related genes and genes upstream of the p53 signaling pathway. Among the 15 genes, cell cycle-related genes accounted for the largest number (6 genes), suggesting that they might have an important role on the classification of the two clusters. The four genes upstream of the p53 signaling pathway were *CDKN2A*, *CHEK1*, *CHEK2*, and *GORAB*. Although *CDKN2A* regulates p53 expression via *MDM2*, it receives repression feedback from p53 and an inverse correlation between *CDKN2A* expression and p53 function has been reported in human tumor cell lines^52^. *CHEK1* phosphorylates p53 at multiple sites, while also receiving feedback from p53 to repress its expression^53^. Furthermore, *GORAB* inhibits p53 ubiquitination via *MDM2* and receives feedback from p53 to repress its expression^54^. Based on these reports, we hypothesized that this feedback was the basis for why upstream genes of p53 were extracted in this study. *CDKN2A* encodes two transcripts (p16INK4a and p14ARF) with different transcription start sites^55^. p16INK4a inhibits cell cycle progression by directly blocking the interaction between CDK4/6 and cyclin D^56^. p14ARF inhibits p53 degradation through direct binding to MDM2^57^. Both CHK1 and CHK2 encoded by *CHEK1* and *CHEK2*, respectively, inhibit the activation of CDC25, an important factor in G2/M phase progression^58^. Furthermore, SCYL1BP1, encoded by *GORAB*, promotes MDM2 degradation and inhibits cell cycle progression in the G1/S phase^59^. Therefore, all of the four abovementioned genes have inhibitory functions on the cell cycle, suggesting that the cell cycle might have a particularly large impact on the classification of the two clusters among the functions of p53 signaling pathway. Additionally, *TP53* mutations reportedly accelerate cell cycle pathways in breast cancer and lung adenocarcinoma, which were classified as cluster A1 in this study^60^. Contrarily, the acceleration of cell cycle pathways by *TP53* mutations was not observed in colon cancer and squamous cell carcinoma of the head and neck, which were classified as cluster A2^60^. These reports support the validity of the results obtained in this study. Based on the abovementioned points, we examined the changes in the expression of cell cycle-related genes on the p53 signaling pathway due to *TP53* mutations between clusters A1 and A2. Altered expression levels were observed more in the cell cycle-related genes in cluster A2 than in cluster A1, suggesting that the changes in cell proliferative activity might be particularly important as a phenotypic difference caused by *TP53* mutations between clusters A1 and A2.

Ki-67 has been reported as a marker of cell proliferative activity in various cancer types^61–63^. *MKI67*, the gene encoding Ki-67, is located on chromosome 10q25. *MKI67* is expressed throughout the cell cycle except during the G0 phase^64^. The expression of *MKI67*, as well as Ki-67, is known as a cell proliferative marker in various cancer types^44,45^. Our results showed that, in many cancer types of cluster A1, the expression level of *MKI67* was significantly higher in the *TP53* mutant group than in the *TP53* wild-type group. Contrarily, in most cancer types in cluster A2, the expression of *MKI67* did not differ between the *TP53* mutant and *TP53* wild-type groups. From this result, the cell proliferative activity in the *TP53* mutant group might be increased as compared to that of the *TP53* wild-type group in cluster A1, which had relatively large changes in expression of cell cycle-related genes on the p53 signaling pathway due to the *TP53* mutations. Contrarily, the cell proliferative activity in the *TP53* mutant group might be less changed compared to the *TP53* wild-type group in cluster A2, which had relatively small changes in the expression of cell cycle-related genes due to the *TP53* mutations.

It has been reported that the expression status of *MKI67* positively correlates with tumor growth and malignancy^65^. Several retrospective analyses have examined the prognostic significance of *MKI67* expression status and reported that a high expression of *MKI67* in cancer tissues was associated with early recurrence after radical resection and high malignancy of the tumor^66–68^. *MKI67* expression level correlated with the sensitivity to anticancer drugs in several cancer types, such as breast^69^ and ovarian cancers^70^. Based on the results of this study and these findings, we hypothesized that *TP53* mutations in cancer types of cluster A1 increase tumor malignancy and shorten the prognosis but they increase sensitivity to anticancer drugs compared to cancer types of cluster A2. We tried to validate the association between *TP53* mutations and prognosis, including response to chemotherapy, in the TCGA cohort, but the validation was difficult because of the insufficient prognostic follow-up period, the extremely small number of cases in many cancer types when matched for case background (age, sex, stage, etc.) between patients with wild-type and mutant *TP53*, and the lack of treatment data (treatment line, drugs, etc.). Instead, we found some reports that supported our findings of the prognosis, such as reports of poor prognosis for the *TP53* mutation group in breast cancer^21,71^ and bladder cancer^72^, and no prognostic impact of *TP53* status in colon cancer and rectal cancer^24,73^. Moreover, we found some reports that supported our findings of the response to chemotherapy, such as reports of *TP53* mutation as a marker of chemotherapy response in breast cancer^31^and bladder cancer^74^, and as a marker of resistance to chemotherapy in head and neck cancer^32,75^. Data from clinical sequencing and the efficacy of chemotherapy and prognosis are now being collected in Japan. We are planning additional studies using these data to validate our hypothesis. The results of this study suggest that the cascade mediated by *TP53* mutations may be responsible for the poor prognosis in cancer types of cluster A1. Therefore, we expect that molecularly targeted therapies reactivating mutant p53 inactivation, such as eprenetapopt for myelodysplastic syndromes and COTI-2 for triple negative breast cancer, may be more sensitive to the cancers in cluster A1 than in cluster A2.Therefore, our study findings were considered to be of great significance in the context of expanding personalized precision medicine based on the information of genetic mutations, including *TP53* genotype, through the introduction of cancer gene panel examinations into clinical practice.

GOF and non-GOF mutations are known to result in loss of function for downstream genes. However, in the first part of this study, we showed that the impact of *TP53* mutations on gene expression in the p53 signaling pathway listed in KEGG among various cancer types, despite the fact that *TP53* mutations (GOF + non-GOF mutations) exhibit loss of function. This suggests that the effects of GOF and non-GOF mutations on the genes of the p53 signaling pathway listed in KEGG may also differ across cancer types; thus, we conducted the latter part of the analysis to examine this point. To date, there have been no reports comparing and validating whether there are differences in effects on the overall p53 pathway between GOFs and non-GOFs across various cancers. This is the first report to examine the impact of distinct *TP53* mutation subtypes (GOF and non-GOF mutations) on the gene expression profile of the p53 signaling pathway through a cross-cancer expression analysis. We found that there was no clear difference between them. The effects of GOF mutations on the expression of genes other than *TP53* have been reported, such as the upregulation of *EGFR* in lung cancer cell lines^76^ and of *HSPG2* in mouse models of pancreatic cancer^77^. Furthermore, genes reported to be associated with GOF, such as *EGF*^36^, *PDGF*^78^, and *VEGF*^79^, which are genes involved in growth factor signaling, and *KLF17*^80^, a negative regulator of metastasis and the epithelial-mesenchymal transition, are not included in the p53 pathway listed in KEGG. This suggests that GOF may have a significant impact on genes other than the classical p53 signaling pathway of KEGG. In addition, GOF and non-GOF mutations reportedly have different phenotypic outcomes. For example, prostate cell lines with GOF mutations have higher cell proliferative activity under androgen deprivation than those with non-GOF mutations^42^. GOF and non-GOF mutations also have different prognostic effects depending on the primary site of colorectal cancer^41^. Our study results showed no distinct difference between GOF and non-GOF mutations in their effects on the changes in the gene expression profile of the p53 signaling pathway. To clarify the mechanism by which different *TP53* mutation subtypes have different phenotypic effects, further studies that combine approaches based on a comparative analysis of comprehensive gene expression profiles, genetic changes due to mutations in tumor suppressor genes and oncogenes other than *TP53*, and epigenetic changes including DNA methylation abnormalities should be required. Furthermore, temperature-sensitive *TP53* mutations have been reported^81^, and an important relationship between *TP53* mutations and host immune responses was found previously^82^. Thus, it will be necessary to study the phenotypic effects of these mutations in the future.

This study has several limitations. First, the case backgrounds, such as sex, age, and tumor stage, were not standardized across cancer types because of the retrospective design of the present study. Second, the use of the median gene expression value for each cancer type enabled a cross-cancer analysis, but the small sample size (21 cancer types in cohort A and 17 cancer types in cohort B) provides insufficient power for statistical analyses. Third, since some cancer types were excluded from this study, we cannot rule out the possibility that some cancer types do not belong to either cluster A1 or A2 obtained in this study. Fourth, although the GOF and non-GOF mutations were defined based on the IARC mutation data, some *TP53* mutations have not yet been considered in terms the GOF mutation characteristics. Therefore, some of the mutations defined as non-GOF mutations in this study may possibly include mutations that are actually GOF mutations. Fifth, other various biological factors may influence the changes in the gene expression profile of the p53 signaling pathway, such as the differences in *TP53* mutation patterns, coexistence of other genetic mutations, and epigenetic changes. And the expression of *TP53* gene itself is also affected by nonsense-mediated mRNA decay (NMD)^83,84^ and loss-of-heterozygosity (LOH)^85,86^ as well as by *TP53* mutations. To conduct these integrated analyses, it is necessary to construct a more large-scale database. Currently, the National Cancer Center and Cancer Genome Information Management Center (C-CAT; Center for Cancer Genomics and Advanced Therapeutics) are continuously collecting cancer genome and clinical information of a vast number of cancer patients. We believe that such a database will enable the integrated analyses described above and will further our understanding of the molecular biological abnormalities of cancers caused by *TP53* mutations.

In conclusion, our findings revealed that the cancer types can be classified into two major groups based on the magnitude of gene expression changes related to the cell cycle and cell proliferative activity caused by *TP53* mutations. Furthermore, there was no distinct difference in the effects of GOF and non-GOF mutations on the gene expression profile of the p53 signaling pathway.

## Methods

### Cases and cancer types

All the data in this study are publicly accessible at the TCGA dataset. This study included all 32 cancer types at the TCGA database (adrenocortical carcinoma, bladder urothelial carcinoma, breast invasive carcinoma, cervical squamous cell carcinoma, colon adenocarcinoma, esophageal carcinoma, glioblastoma multiforme, head and neck squamous cell carcinoma, kidney chromophobe, brain lower grade glioma, liver hepatocellular carcinoma, lung adenocarcinoma, lung squamous cell carcinoma, mesothelioma, ovarian serous cystadenocarcinoma, pancreatic adenocarcinoma, prostate adenocarcinoma, rectum adenocarcinoma, sarcoma, stomach adenocarcinoma, uterine corpus endometrial carcinoma, uterine carcinosarcoma, skin cutaneous melanoma, cholangiocarcinoma, diffuse large B-cell lymphoma, thymoma, kidney renal papillary cell carcinoma, kidney renal clear cell carcinoma, testicular germ cell tumors, thyroid carcinoma, pheochromocytoma and paraganglioma, and uveal melanoma). We collected the gene expression data, clinical information, and *TP53* genotype of all 32 cancer types from cBioportal (https://www.cbioportal.org/). This study was conducted in accordance with the Declaration of Helsinki.

### Comparison of p53 transcriptional activity

IARC database has reported all transcriptional activities of 2,314 different mutant p53 relative to p21, MDM2, BAX, 14-3-3σ, AIP1, GADD45, NOXA and p53R^19^. For p21 and each other target gene, we calculated the correlation coefficients for the transcriptional activity of 2,314 different mutant p53.

### Definition of TP53 genotype

As of September 2021, we obtained *TP53* mutation data by Whole Exome Sequencing from cBioportal. Among the*TP53* gene mutations, only mutations with an allele frequency of > 20% were recognized as mutations^87^. We defined “*TP53* mutation” as nonsense and frameshift mutations of *TP53* gene. Missense mutations that were reported to reduce the transcriptional activity of p53 on p21 in the IARC database were also defined as “*TP53* mutation,” and cases with the other missense mutations were excluded from this study. The absence of mutations in the *TP53* gene was defined as “*TP53* wild-type.” Among *TP53* mutations, we defined “hotspot mutation” as those that corresponded to six hot spot locations (R175, G245, R248, R249, R273 and R282)^4^. The proportion of hotspot mutation is the ratio of the number of hotspot mutations in the total number of mutations. IARC defines GOF mutation as a functional property that mutant p53 but not wild-type p53 exhibits by measuring the activity of proteins overexpressed in human and yeast cells. Among the *TP53* mutations, the mutations reported as GOF *TP53* mutations in the IARC database were defined as “GOF mutation” and the mutations other than GOF mutations were defined as “non-GOF mutation.” All variants of *TP53* mutation were included in the comparing GOF and non-GOF mutations.

### Cohort for analyses

From 32 cancer types, the group of cancer types contained 10 or more cases each with *TP53* mutations, and *TP53* wild-type cases were categorized into “cohort A.” Among the cancer types in cohort A, the group of cancer types containing 10 or more cases of GOF and non-GOF mutations each was defined as “cohort B”.

### Genes on the p53 signaling pathway

Genes on the p53 signaling pathway in KEGG were used for this study. Gene expression data registered with TCGA were obtained by RNA sequencing. As of September 2021, Fragments Per Kilobase of exon per Million mapped reads (FPKM) normalized data were obtained from the Genomic Data Commons Data Portal (GDC Portal, https://portal.gdc.cancer.gov/) and converted into TPM for analysis. The median expression levels of each gene on the p53 pathway were calculated for each cancer type. Genes with the median expression value of < 1.0^43^ in more than half of the cases in cohort A or B were excluded from further analyses.

### Cross-cancer unsupervised hierarchical cluster analyses based on gene expression profile changes

The effects of *TP53* mutations on the gene expression profile of the p53 signaling pathway can be expressed as the gene expression ratio of the median expression value of *TP53* mutant group to that of the *TP53* wild-type group. Therefore, in cohort A, we used the logarithm of the expression ratio of each gene with a base of 2 (Log2 [median gene expression of *TP53* mutant group]/[median gene expression of *TP53* wild-type group]) for a cross-cancer unsupervised hierarchical cluster analysis. This cluster analysis was performed using the complete linkage method with Pearson correlation distance by using the Multiple Experiment Viewer (MeV 4.9.0, National Library of Medicine of the US National Institute of Health, http://mev.tm4.org/). Similarly, in cohort B, the logarithm of the expression ratio of GOF to *TP53* wild-type for each gene with a base of 2 (Log2 [median gene expression of GOF mutant group]/[median gene expression of *TP53* wild-type group]) and of non-GOF to *TP53* wild-type (Log2 [median gene expression of non-GOF mutant group]/[median gene expression of *TP53* wild-type group]) were used for a cross-cancer unsupervised hierarchical cluster analysis.

### Identification of factors contributing to clustering

When comparing the characteristics between the clusters in the cohort A, histologic types were classified into adenocarcinoma and non-adenocarcinoma, and the developmental origin into endoderm, mesoderm, and ectoderm, based on a previous report^88^, and their proportions were compared.

Additionally, the proportion of *TP53* and GOF mutant cases for each cancer type were compared between the clusters. The genes with significantly different expression ratios (*P* < 0.05 by Wilcoxon rank sum test) between the clusters were also extracted.

### Statistical analysis

Statistical analysis was performed using R’s ExactRankTests package or JMP Pro16® (SAS Institute, Cary, NC). The Pearson correlation coefficient was used to calculate the correlation coefficient. Wilcoxon rank sum tests were used to examine the differences in the proportion of cases between the two groups, comparison of gene expression values, extraction of genes with differential expression values, and comparison of the number of genes extracted. Fisher’s exact test was used to compare the characteristics of cancer types among the clusters and the number of applicable cancer types among the clusters. The significance levels were set at *P* < 0.05.

## Supplementary Information

Supplementary Information.

## Data Availability

Gene expression and *TP53* mutation data of each patient used in this study can be retrieved from the public GDC Portal (https://portal.gdc.cancer.gov/) and cBioportal (https://www.cbioportal.org/), respectively.
